# Supplementary material for: A new approach exploiting thermally activated delayed fluorescence molecules to optimize solar thermal energy storage
Source: Nat Commun. 2022 Feb 10;13:797. doi: 10.1038/s41467-022-28489-0 (PMC8831622; doi:10.1038/s41467-022-28489-0)
Supplement: Supplementary file 1 — Supplementary Information [file 41467_2022_28489_MOESM1_ESM.pdf]

Supplementary Information

A New Approach Exploiting Thermally Activated Delayed Fluorescence Molecules to Optimize Solar Thermal Energy Storage

Fan-Yi Meng,[1,2] I-Han Chen,[1,2] Jiun-Yi Shen,[1,2] Kai-Hsin Chang,[1,2] Tai-Che Chou,[1] Yi-An Chen,[1] Yi-Ting Chen,[1] Chi-Lin Chen,[1] Pi-Tai Chou[1,*]

[1]Department of Chemistry, National Taiwan University, Taipei, 10617 Taiwan, R.O.C.

[2]These authors contributed equally

Table of Contents

1. Synthesis Route and Procedures S2

2. Photophysical Measurements in Solution State S12

3. Photochemistry and Isomerization S18

4. Photophysical Measurements in Solid State S23

5. *Supplementary Reference* S26

1. Synthesis Route and Procedures

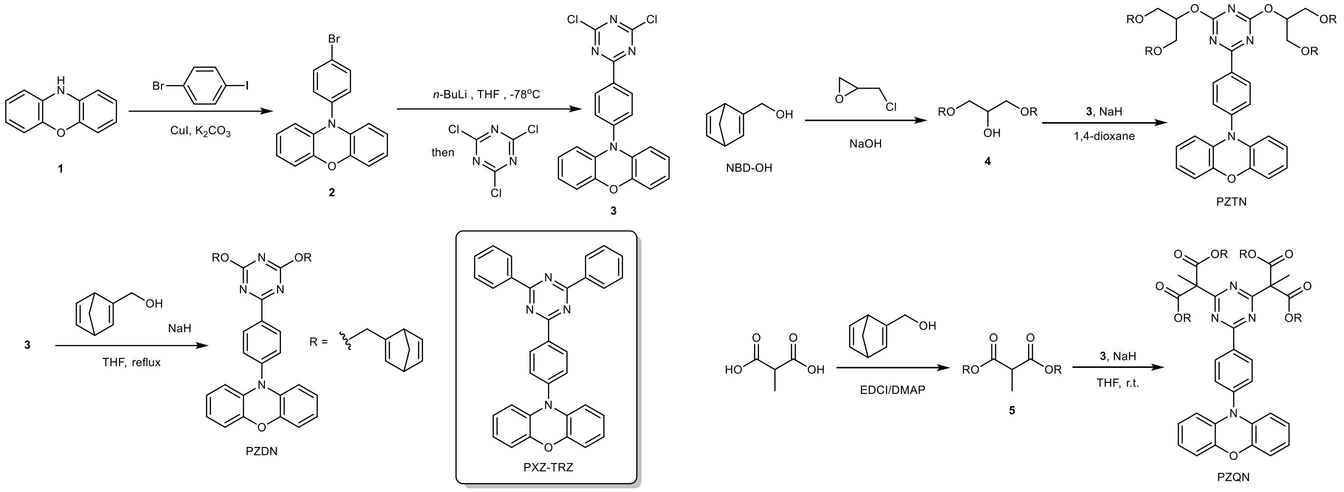

Supplementary Figure 1. The synthetic route of PZDN, PZTN and PZQN.

Synthesis of Compound 2 and 3. We started from phenoxazine that reacted with 4-iodo-bromobenzene, potassium carbonate, copper(I) iodide to obtain compound **2**. Next, a solution of **2** (2.0 g, 5.91 mmol) in 40 mL of anhydrous THF was cooled to -78 °C under a nitrogen atmosphere and was treated dropwise with 2.5 M n-BuLi in hexane (2.84 mL, 7.1 mmol). The resulting mixture was stirred at -78 ℃. After 1 h, the mixture was quickly added to a solution of cyanuric chloride (5.45 g, 29.6 mmol) in 40 mL of THF at 0 ℃. The resulting mixture was stirred at room temperature overnight. The reaction was quenched with water and extracted with CH_2Cl_2. The combined organic layers were dried over $MgSO_4$, filtered and evaporated. The crude product was purified by silica gel column chromatography with Hexane/CH_2Cl_2 mixture as eluent to afford **3** (0.98 g, 40%) as deep purple solids.

^{1}H NMR (400 MHz, $CDCl_3$): δ 6.01 (d, *J* = 8.4 Hz, 2 H), 6.61-6.75 (m, 6 H), 7.54 (d, *J* = 8.6 Hz, 2 H), 8.71 (d, *J* = 8.6 Hz, 2 H).

Synthesis of Compound PZDN. A solution of bicyclo [2.2.1]hepta-2,5-diene-2-methanol (300 mg, 2.5 mmol) in 10 mL of anhydrous THF was added to 60% NaH (94 mg, 2.4 mmol) in 10 mL anhydrous THF under nitrogen atmosphere. The resulting mixture was stirred at RT. After 1 h, the mixture was added dropwise to a solution of **3** (250 mg, 0.61 mmol) in 10 mL of THF. The resulting mixture was stirred at room temperature overnight. The reaction was quenched with water and extracted with CH_2Cl_2. The combined organic layers were dried over $MgSO_4$, filtered and evaporated. The crude product was purified by silica gel column chromatography with Hexane/CH_2Cl_2 mixture as eluent to afford PZDN (245 mg, 70%) as bright yellow solids.

^{1}H NMR (400 MHz, C_6D_{12}): δ 2.03 (d, *J* = 6 Hz, 2 H), 2.08 (d, *J* = 6 Hz, 2 H), 3.52 (s, 4 H), 5.06 (s, 4 H), 5.91 (d, *J* = 8 Hz, 2 H), 6.46-6.60 (m, 8 H), 6.67 (m, 2 H), 6.76 (m, 2 H), 7.35 (d, *J* = 8.6 Hz, 2 H), 8.69 (d, *J* = 8.6 Hz, 2 H).

^{13}C NMR (100 MHz, $CDCl_3$): δ 50.4, 51.4, 66.9, 73.8, 113.3, 115.6, 121.6, 123.2, 130.8, 131.7, 133.8, 135.3, 140.5, 142.5, 143.1, 143.4, 143.9, 152.2, 172.4, 173.9.

HR-MS(ESI) *m/z* calcd. for $C_{37}H_{30}N_4O_3$: 578.2312 ([M]$^+$), found: 578.2304.

Compound PZDQC. ^{1}H NMR (400 MHz, C_6D_{12}): δ 1.42-1.56 (m, 6 H, calcd.), 1.67-1.75 (m, 4 H), 1.96-2.11 (m, 4 H), 4.45 (d, *J* = 12.2 Hz, 2 H), 4.71 (d, *J* = 12.2 Hz, 2 H), 5.91 (d, *J* = 8 Hz, 2 H), 6.43-6.59 (m, 6 H), 7.34 (d, *J* = 7.2 Hz, 2 H), 8.70 (d, *J* = 7.2 Hz, 2 H).

Synthesis of Compound 4. A solution of bicyclo[2.2.1]hepta-2,5-diene-2-methanol (1.0 g, 8.2 mmol) in hexane (2 mL) was added a mixture of 18 M NaOH aqueous solution (1 mL) and tetrabutylammonium bromide (32 mg, 0.1 mmol). Under vigorous stirring, epichlorohydrin (303 mg, 3.3 mmol) was added dropwise, then the mixture was heated for 24 h. After cooling to room temperature, saturated aqueous NH_4Cl was added for neutralization, extracted with ether and dried with $MgSO_4$. The crude product was purified through silica gel column chromatography with CH_2Cl_2/MeOH = 100/1 as eluent to afford **4** (650 mg, 66% yield) as a mixture of stereoisomers.

^{1}H NMR (400 MHz, $CDCl_3$): δ 1.96-2.04 (m, 4 H), 2.55 (s, 1 H), 3.30-3.45 (m, 6 H), 3.55 (s, 2 H), 3.93 (p, *J* = 5.4 Hz, 1 H), 4.08-4.20 (m, 4 H), 6.45-6.46 (m, 2 H), 6.73-6.78 (m, 4 H).

^{13}C NMR (100 MHz, $CDCl_3$): δ 50.2, 51.2, 69.5, 69.8, 70.9, 73.6, 138.66, 138.68, 142.4, 142.5, 143.3, 154.6.

HR-MS(ESI) *m/z* calcd. for $C_{19}H_{24}O_3Na$: 323.1618 ($[M + Na]^+$), found: 323.1616.

Synthesis of Compound PZTN. A solution of **4** (790 mg, 2.6 mmol) in anhydrous 1,4-dioxane (10 mL) was added dropwise to a mixture of 60% NaH (94 mg, 2.4 mmol) and 1,4-dioxane (5 mL) at 0 ℃ and stirred for 1 h. **3** (452 mg, 1.05 mmol) in anhydrous 1,4-dioxane (10 mL) was added dropwise, and the mixture was allowed to reflux for 24h. After cooling to RT, saturated aqueous NH_4Cl was added for neutralization, extracted with ether and dried with $MgSO_4$. The crude product was purified through silica gel column chromatography with CH_2Cl_2 as eluent to afford PZTN (705 mg, 72% yield) as a mixture of stereoisomers.

^{1}H NMR (400 MHz, C_6D_6): δ 1.91-2.03 (m, 8 H), 3.32 (s, 4 H), 3.41 (s, 4 H), 3.61-3.76 (m, 8 H), 4.00-4.01 (m, 8 H), 5.80-5.88 (m, 2 H), 5.91 (d, *J* = 9.4 Hz, 2 H), 6.34 (s, 4 H), 6.44 (td, *J* = 7.7, 1.5 Hz, 2 H), 6.51 (td, *J* = 7.7, 1.5 Hz, 2 H), 6.60-6.64 (m, 4 H), 6.74-6.80 (m, 6 H), 6.92 (d, *J* = 8.5 Hz, 2 H), 8.63 (d, *J* = 8.5 Hz, 2 H).

^{13}C NMR (100 MHz, C_6D_6) δ 50.1, 52.0, 69.05, 69.08, 70.4, 74.20, 74.24, 76.5, 114.2, 116.3, 122.3, 124.0, 131.4, 132.4, 134.8, 136.2, 139.1, 143.1, 143.2, 143.7, 143.8, 144.9, 155.4, 155.5, 173.6, 174.8.

HR-MS(ESI) *m/z* calcd. for $C_{59}H_{58}N_4O_7Na$: 957.4198 ($[M + H]^+$), found: 957.4190.

Compound PZTQC. ^{1}H NMR (400 MHz, C_6D_{12}): δ 1.28 (m, 4 H), 1.37-1.42 (m, 4 H, calcd.), 1.49 (m, 4 H), 1.55-1.58 (m, 8 H), 1.93 (d, *J* = 10.6 Hz, 4 H), 2.04 (d, *J* = 10.6 Hz, 4 H), 3.48-3.55 (m, 4 H), 3.68-3.84 (m, 12 H), 5.55 (m, 2 H), 5.94 (d, *J* = 8.2 Hz, 2 H), 6.46-6.61 (m, 6 H), 7.36 (d, *J* = 8.2 Hz, 2 H), 8.71 (d, *J* = 8.2 Hz, 2 H).

Synthesis of Compound 5. A solution of 2-methylmalonic acid (1.18 g, 10 mmol), **3** (3.05 g, 25 mmol) and 4-dimethylaminopyridine (10 mg) in anhydrous dichloromethane (50 mL) was added N-(3-dimethylaminopropyl)-N'-ethylcarbodiimide hydrochloride (5.75 g, 30 mmol). And the mixture was stirred at room temperature overnight. After removed the solvent in vacuo, the residue was purified through silica gel column chromatography with Hexane/CH_2Cl_2 = 1/2 as eluent to afford **5** (1.76 g, 54% yield) as colorless oil.

^{1}H NMR (400 MHz, $CDCl_3$): δ 1.42 (d, *J* = 7.6 Hz, 3 H), 2.00 (ABq, *J* = 6 Hz, 4 H), 3.41 (s, 2 H), 3.46 (q, *J* = 7.6 Hz, 1 H), 3.55 (s, 2 H), 4.77 (m, 4 H), 6.52 (d, *J* = 1.6 Hz, 2 H), 6.72-6.77 (m, 4 H).

^{13}C NMR (100 MHz, $CDCl_3$): δ 13.7, 46.1, 50.2, 51.27, 51.32, 63.8, 73.74, 73.75, 139.95, 139.96, 140.0, 142.41, 142.44, 143.28, 143.3, 152.05, 152.10, 169.8.

HR-MS(FAB) *m/z* calcd. for $C_{20}H_{23}O_4$: 327.1596 ($[M + H]^+$), found: 327.1598.

Synthesis of Compound PZQN. A solution of **5** (600 mg, 1.84 mmol) in 10 mL of anhydrous THF was added to 60% NaH (60 mg, 1.5 mmol) in 10 mL anhydrous THF under nitrogen atmosphere at 0 ℃. After 1 h, the mixture was added with a solution of **3** (250 mg, 0.61 mmol) in 10 mL THF dropwise. The resulting mixture was stirred at room temperature overnight. The reaction was quenched with water and extracted with CH_2Cl_2. The combined organic layers were dried over $MgSO_4$, filtered and evaporated. The crude product was purified by silica gel column chromatography with Hexane/CH_2Cl_2 = 1/2 as eluent to afford PZQN (413 mg, 69%) as orange oil.

^{1}H NMR (400 MHz, $CDCl_3$): δ 1.95 (ABq, *J* = 6 Hz, 8 H), 2.00 (s, 6 H), 3.37 (s, 4 H), 3.52 (s, 4 H), 4.86 (ABq, *J* = 14.3 Hz, 8 H), 5.95 (d, *J* = 7.8 Hz, 2 H), 6.50 (s, 4 H), 6.57-6.73 (m, 14 H), 7.46 (d, *J* = 8.6 Hz, 2 H), 8.64 (d, *J* = 8.6 Hz, 2 H).

^{13}C NMR (100 MHz, $CDCl_3$) δ 20.6, 50.2, 51.21, 51.23, 63.9, 64.7, 73.72, 73.74, 113.2, 115.7, 121.7, 123.2, 131.0, 132.0, 133.7, 134.7, 140.0, 142.4, 143.27, 143.3, 143.6, 143.9, 151.82, 151.85, 168.7, 170.3, 176.4.

HR-MS(ESI) *m/z* calcd. for $C_{61}H_{54}N_4O_9Na$: 1009.3783 ($[M + H]^+$), found: 1009.3783.

NMR Spectra:

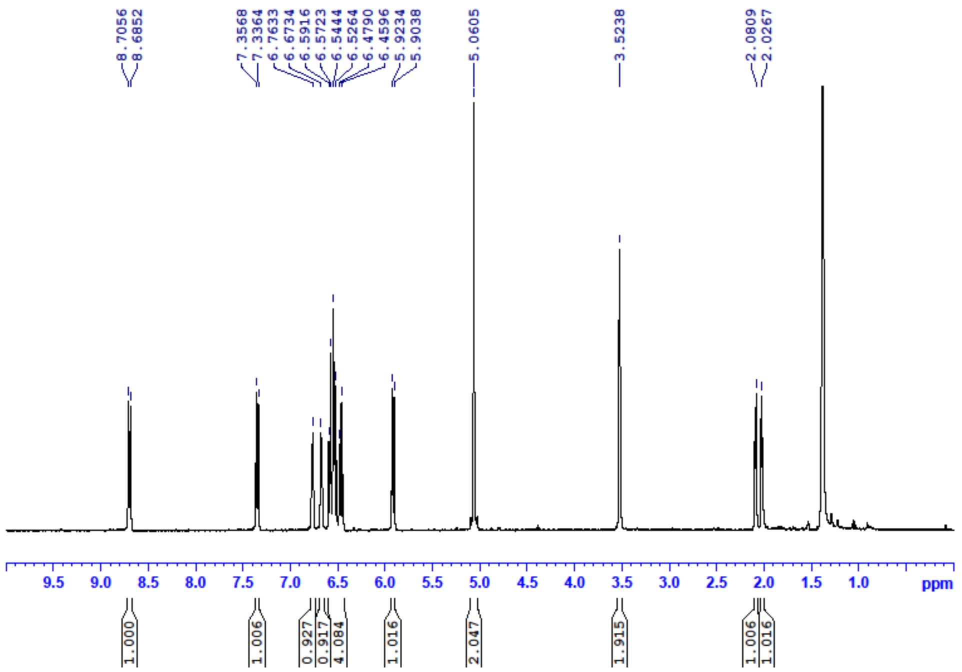

Supplementary Figure 2. **The ^{1}H NMR spectrum of PZDN in C_6D_{12}.**

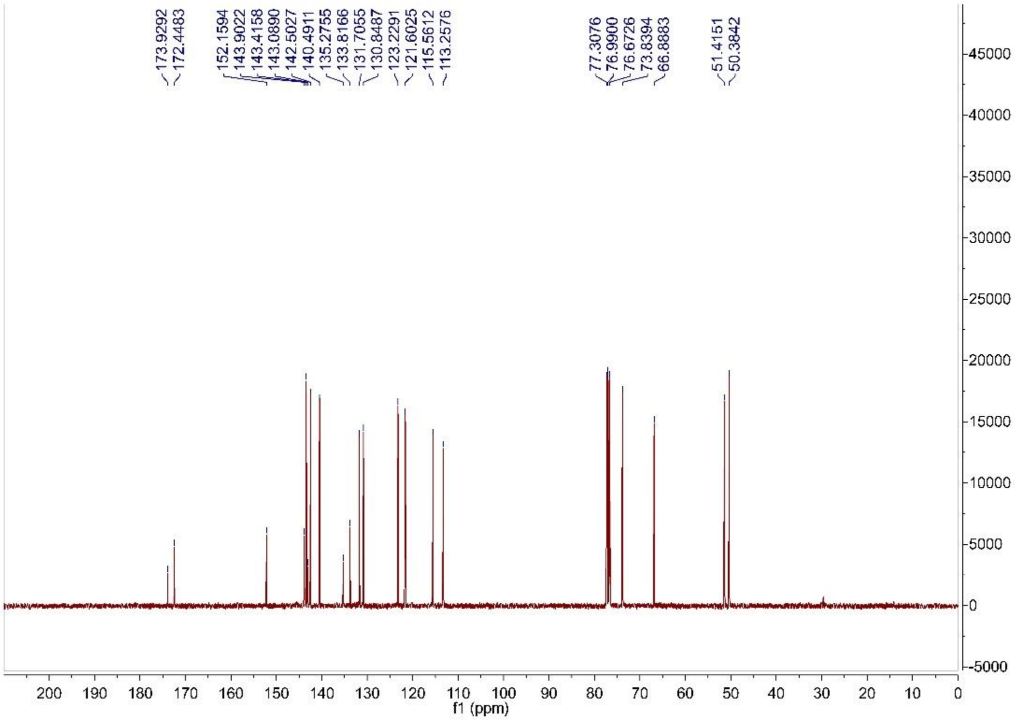

Supplementary Figure 3. **The ^{13}C NMR spectrum of PZDN in $CDCl_3$.**

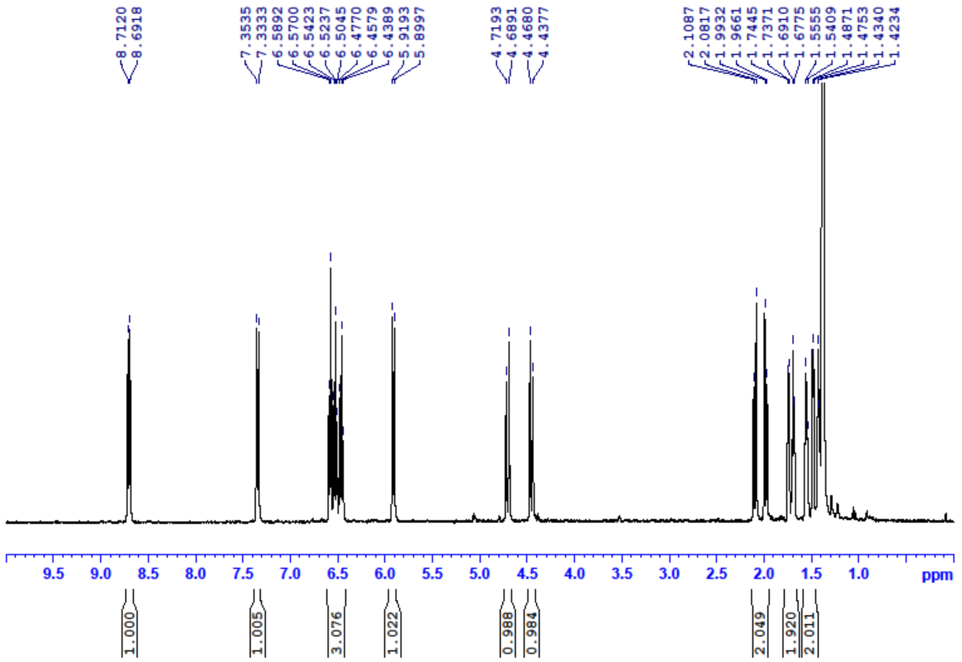

Supplementary Figure 4. The ^1H NMR spectrum of PZDQC in C_6D_{12}.

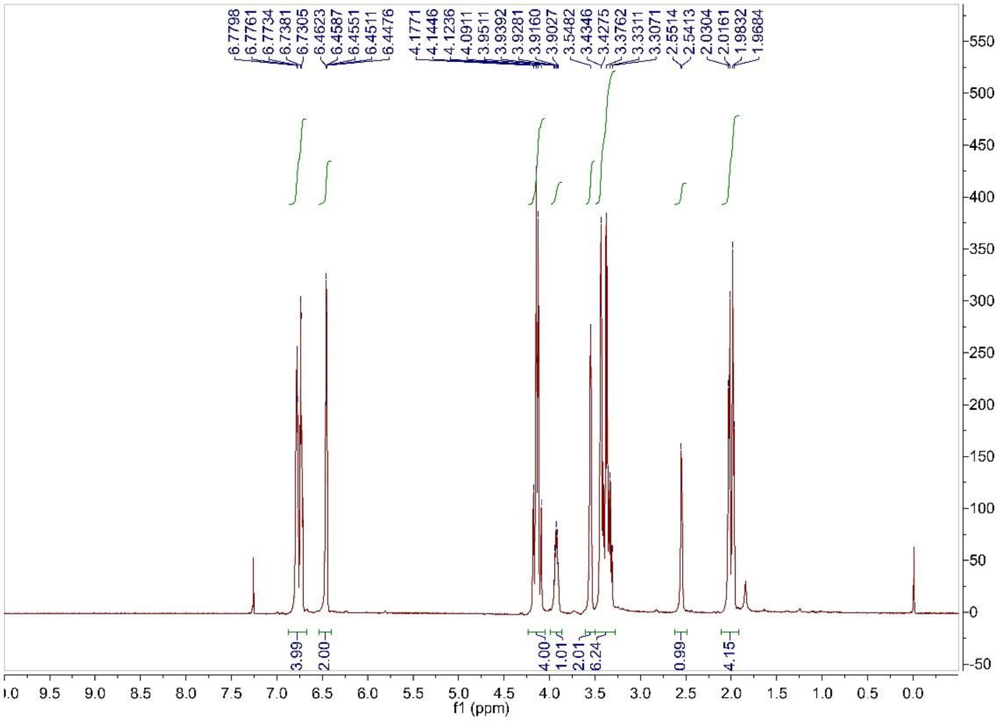

Supplementary Figure 5. The ^1H NMR spectrum of 4 in $CDCl_3$.

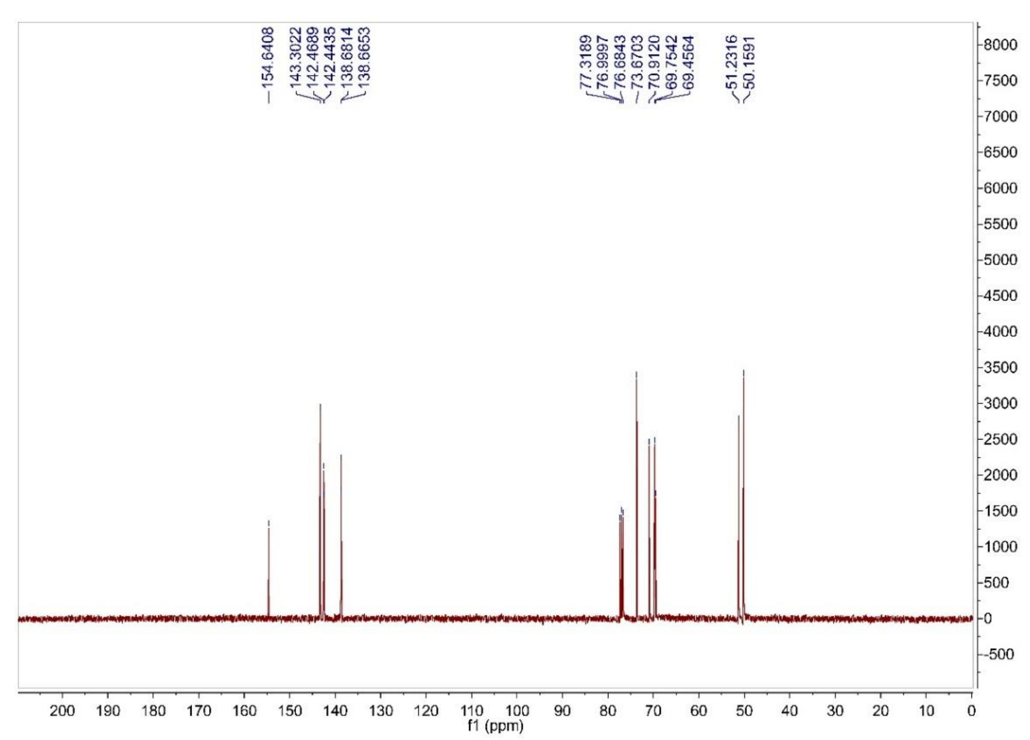

Supplementary Figure 6. **The ^{13}C NMR spectrum of 4 in $CDCl_3$.**

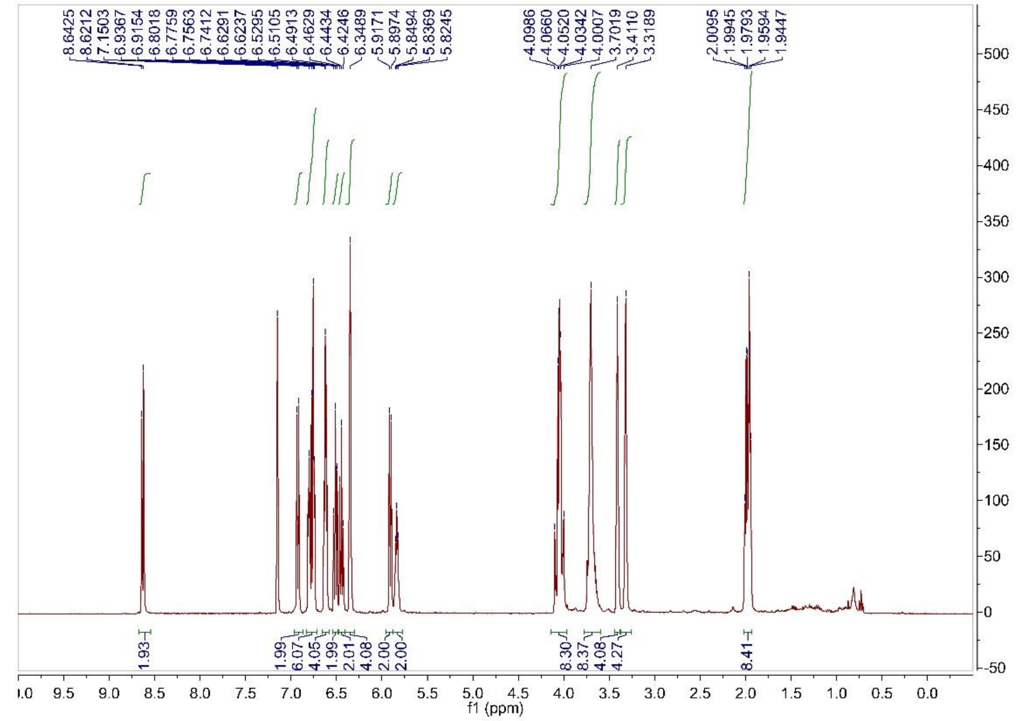

Supplementary Figure 7. **The ^1H NMR spectrum of PZTN in C_6D_6.**

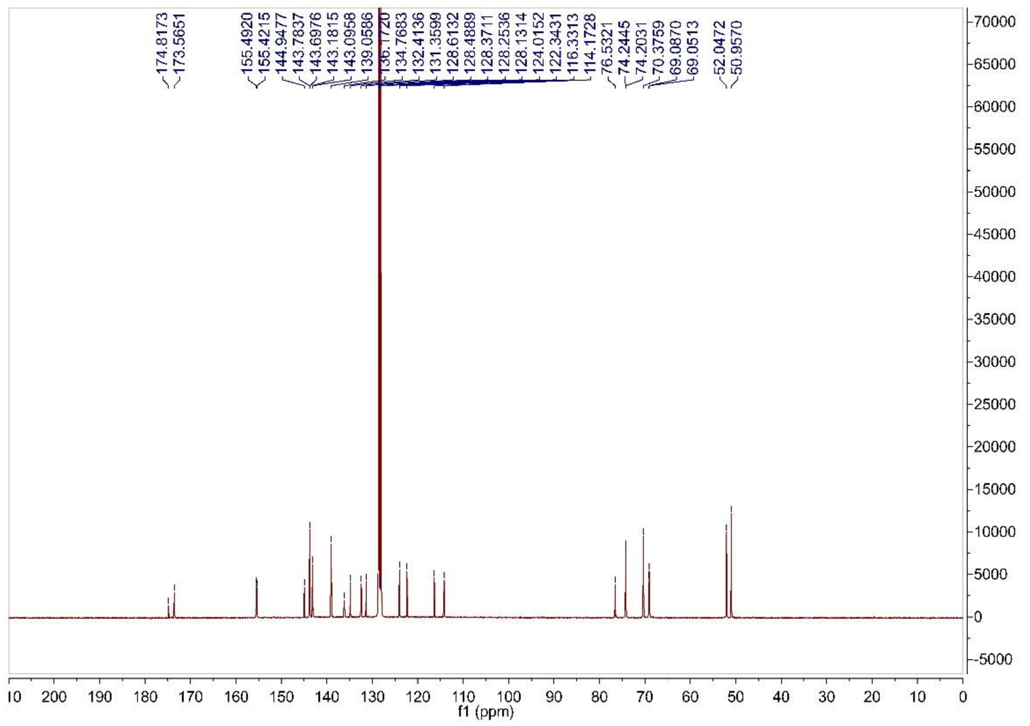

Supplementary Figure 8. **The ^{13}C NMR spectrum of PZTN in C_6D_6.**

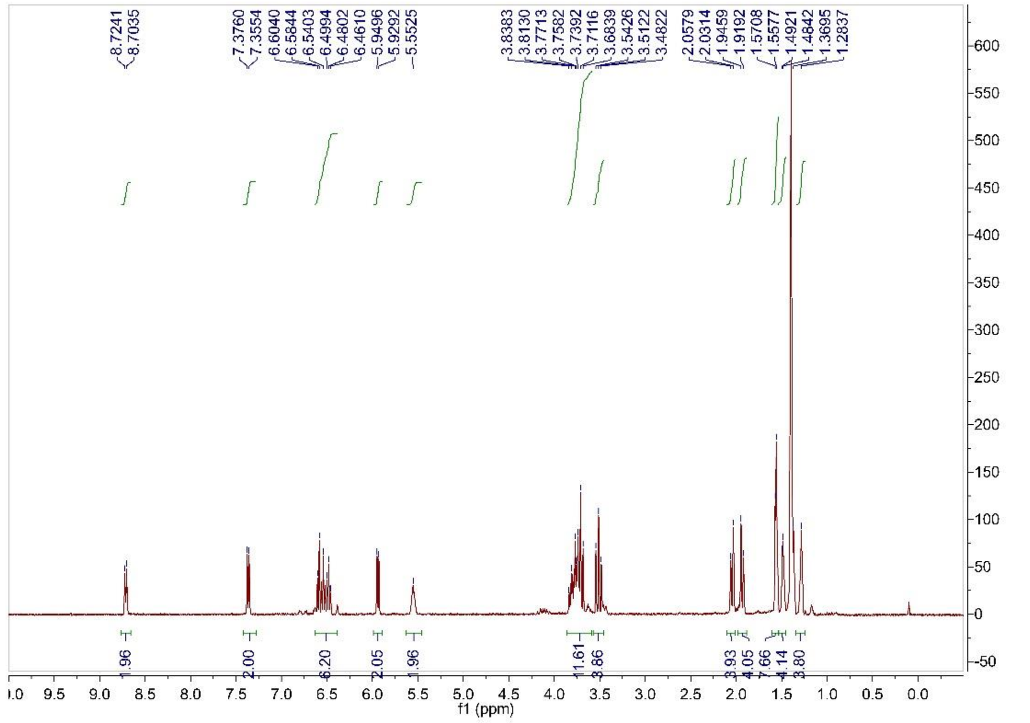

Supplementary Figure 9. **The ^{1}H NMR spectrum of PZTQC in C_6D_{12}.**

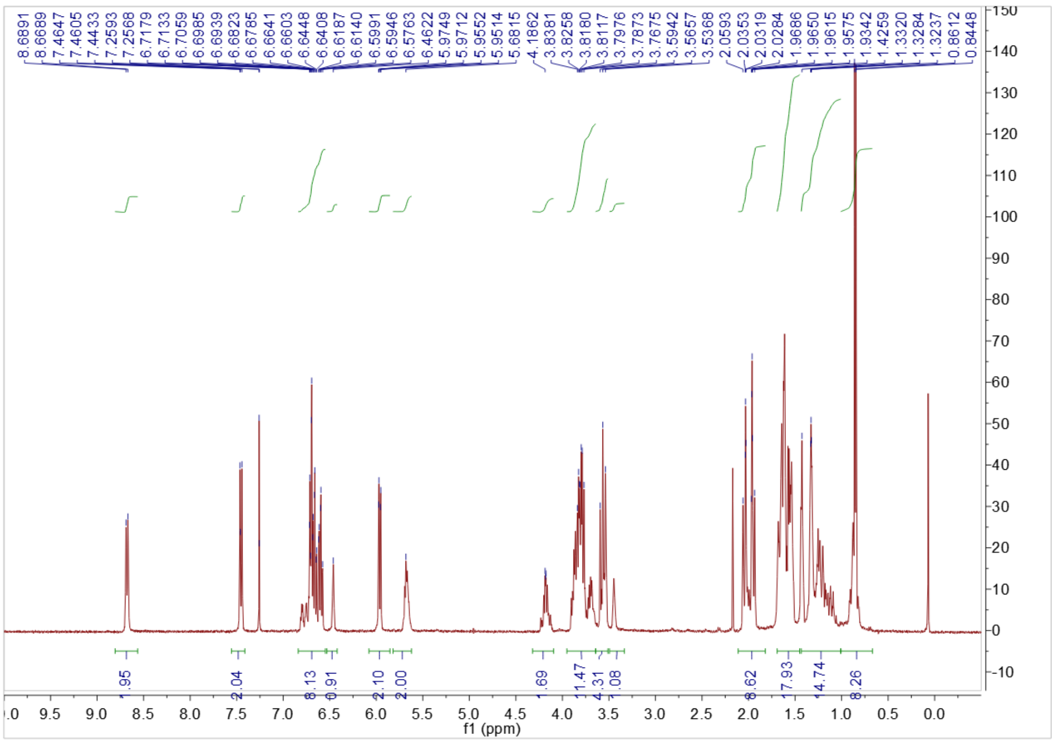

Supplementary Figure 10. **The ^{1}H NMR spectrum of PZTQC in $CDCl_3$, after reflux in methylcyclohexane for 2 h.**

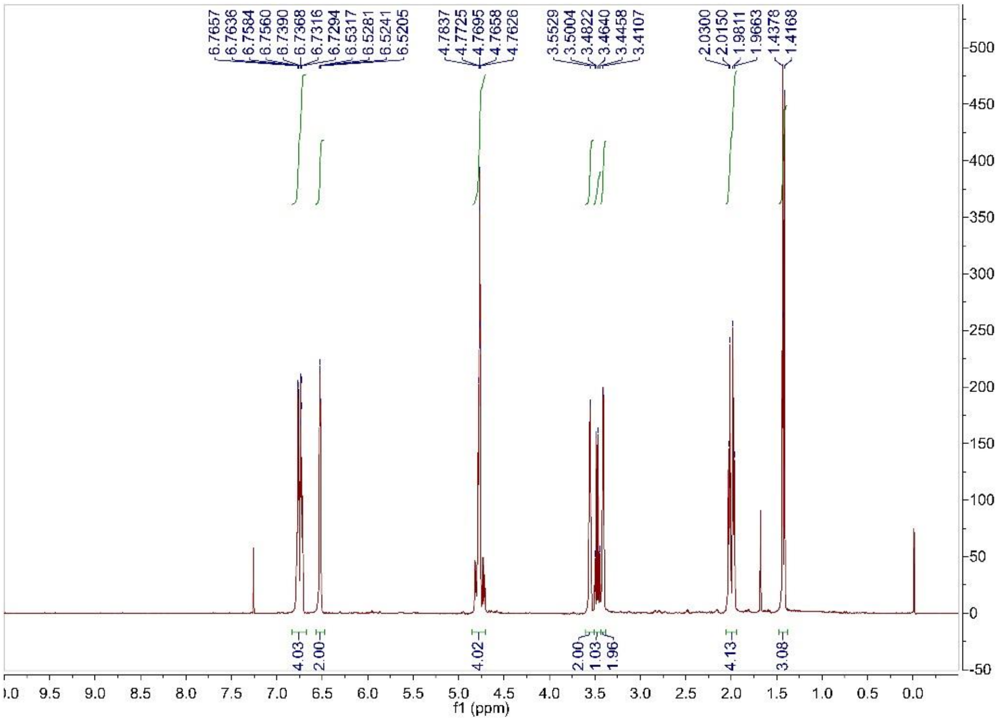

Supplementary Figure 11. **The ^{1}H NMR spectrum of 5 in $CDCl_3$.**

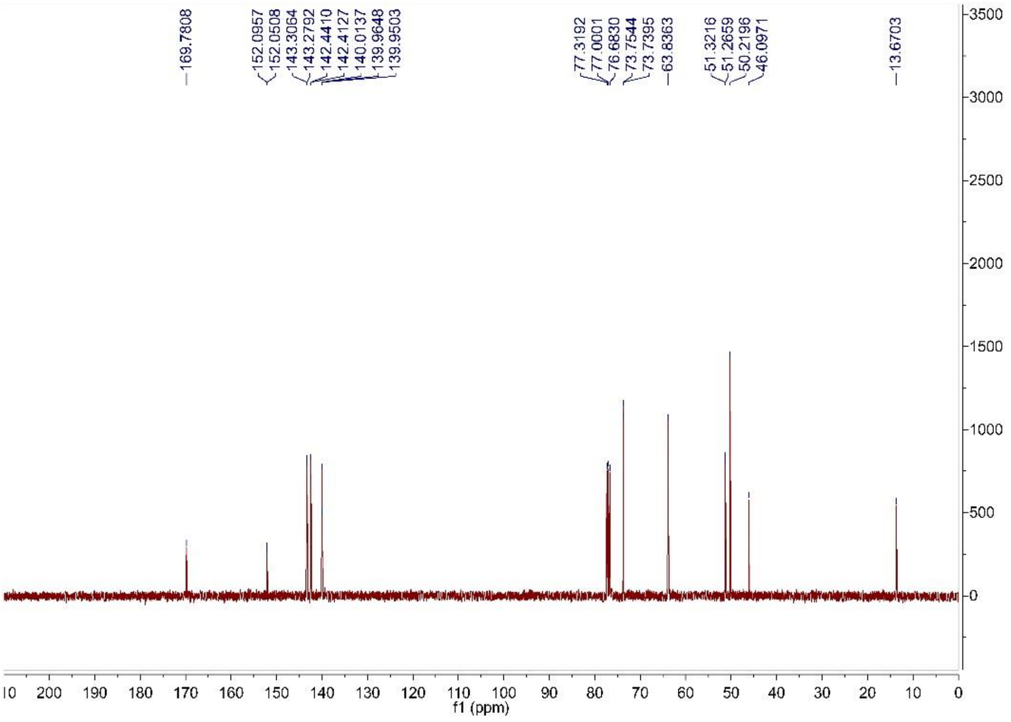

Supplementary Figure 12. **The ^{13}C NMR spectrum of 5 in $CDCl_3$.**

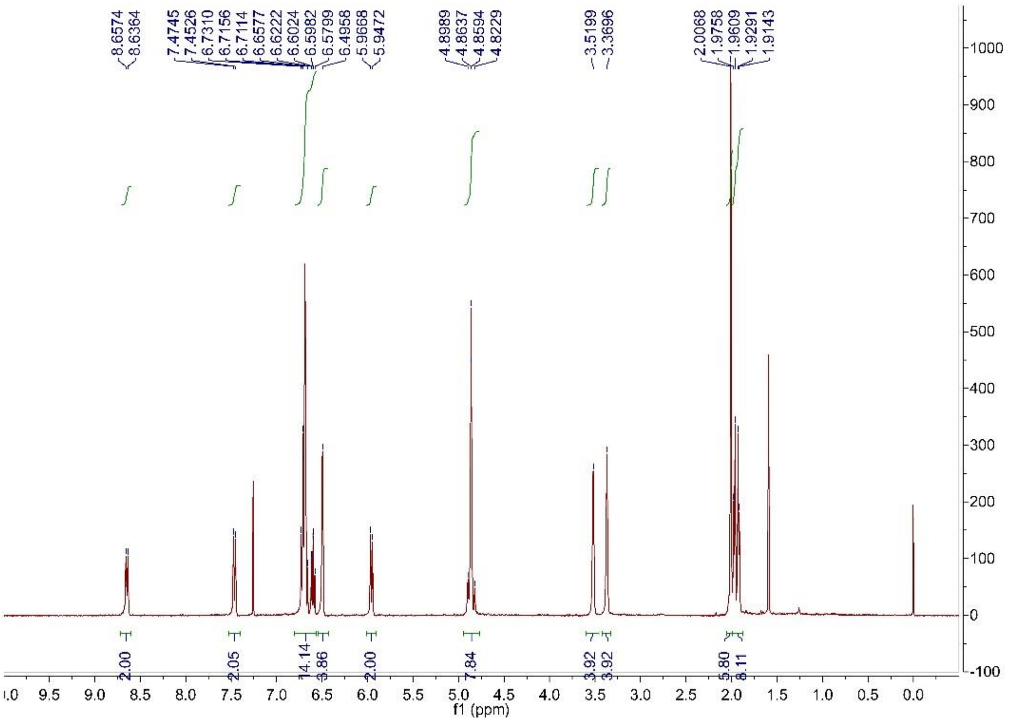

Supplementary Figure 13. **The ^{1}H NMR spectrum of PZQN in $CDCl_3$.**

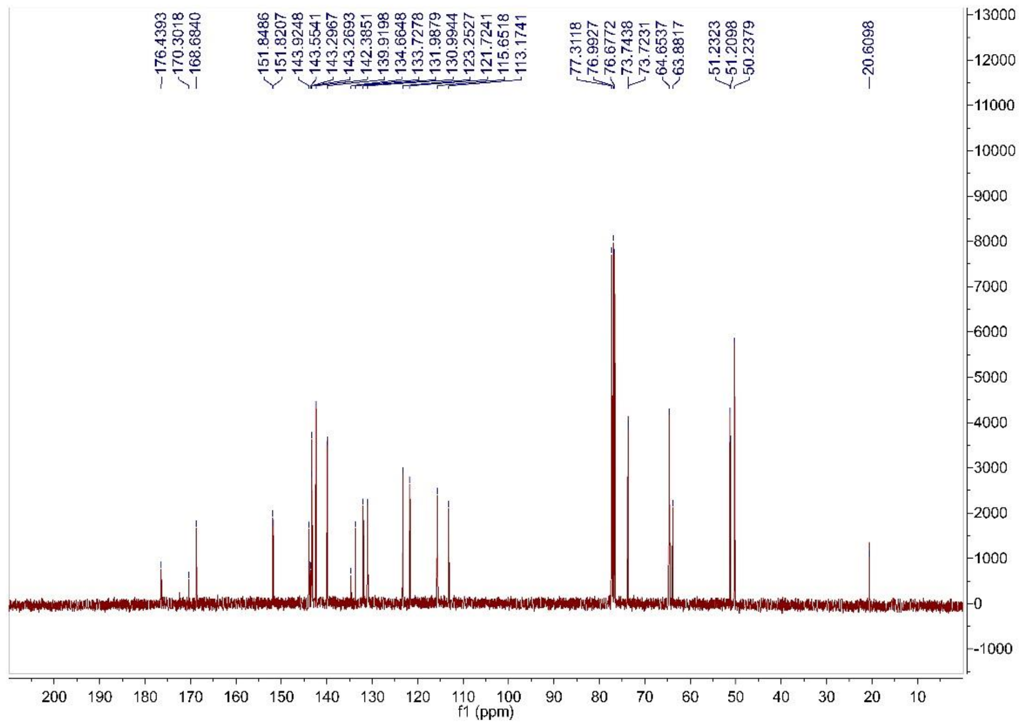

Supplementary Figure 14. **The ^{13}C NMR spectrum of PZQN in $CDCl_3$.**

2. Photophysical Measurements in Solution State

2-1. Excitation spectra

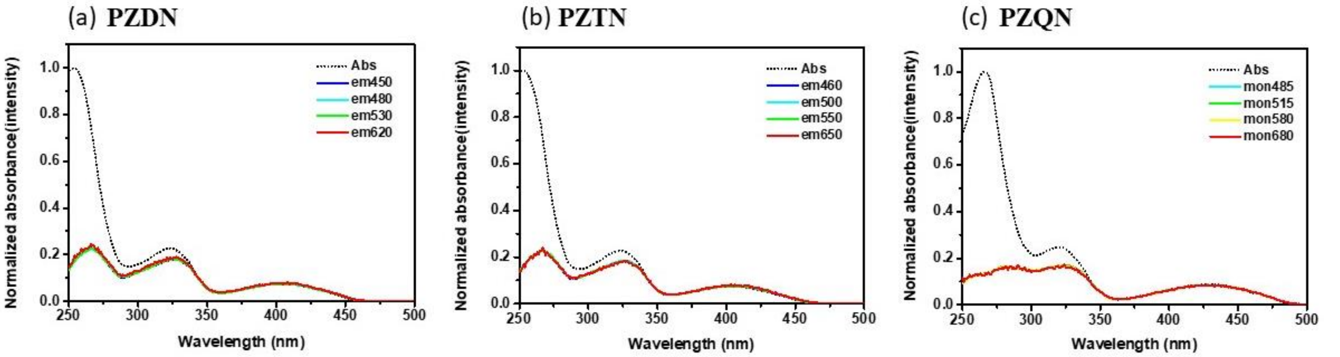

Supplementary Figure 15. The excitation spectra of (a) PZDN, (b) PZDN and (c) PZTN in cyclohexane at room temperature. Note: “em 500” refers to the monitored emission wavelength. Mismatches between absorption and excitation spectra in higher lying region of 250 ~ 350 nm originate from the combination of fast non-radiative decays from highly excited state and inner filter effect.

2-2. Intermolecular triplet-triplet energy transfer

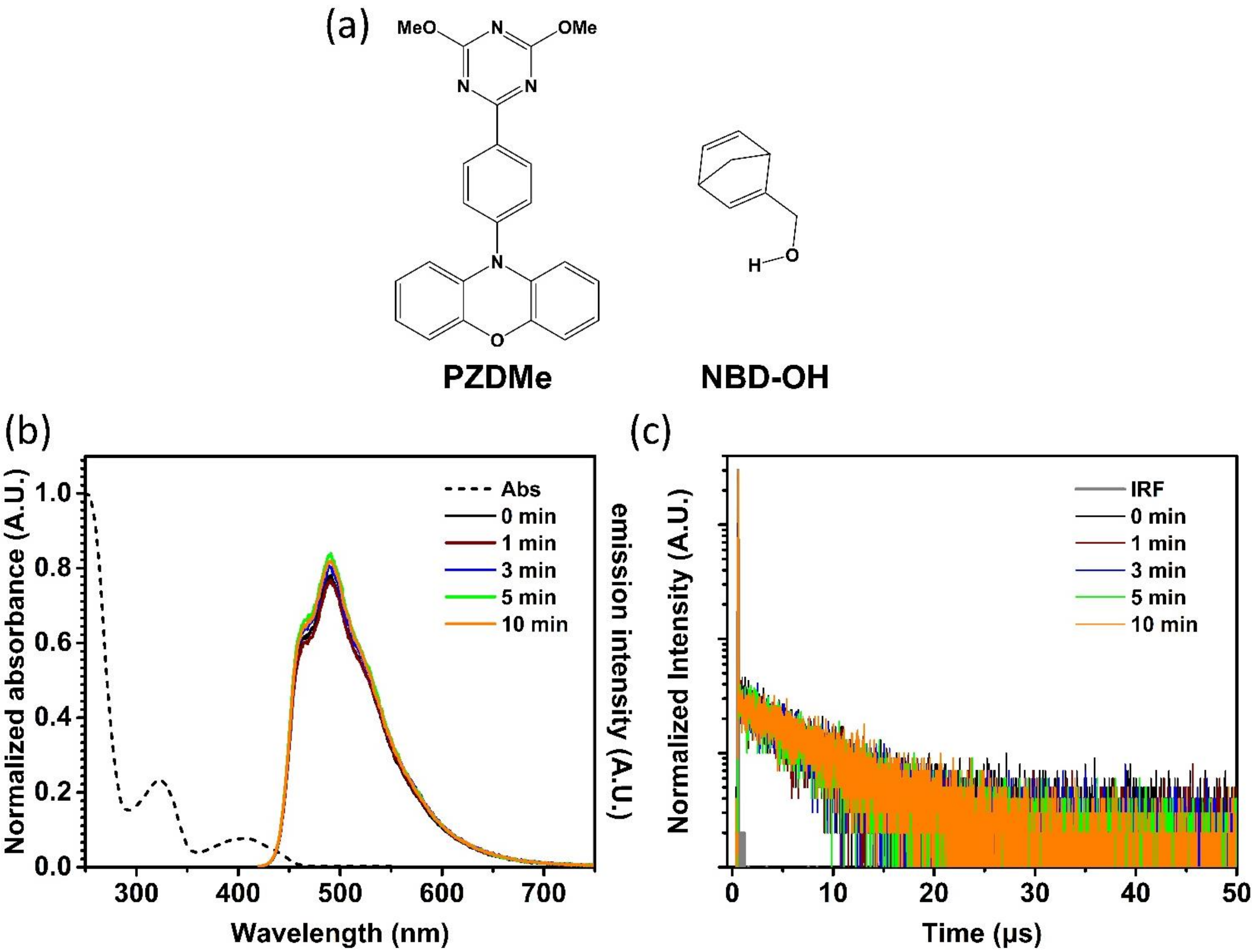

Supplementary Figure 16. Intermolecular sensitization of PZDMe(5×10^{-5} M) and NBD-OH(1×10^{-4} M) mixture in cyclohexane at room temperature. (a) The chemical structure of PZDMe and NBD-OH. (b) Steady state measurements (λ_{ex} = 400 nm) and (c) kinetic trace (excitation wavelength: 378 nm) under degassed condition as a function of irradiation time (λ_{ex} = 405 nm, ~0.1 mW/cm^2).

2-3. Relaxation Dynamics Measurement and Simulation

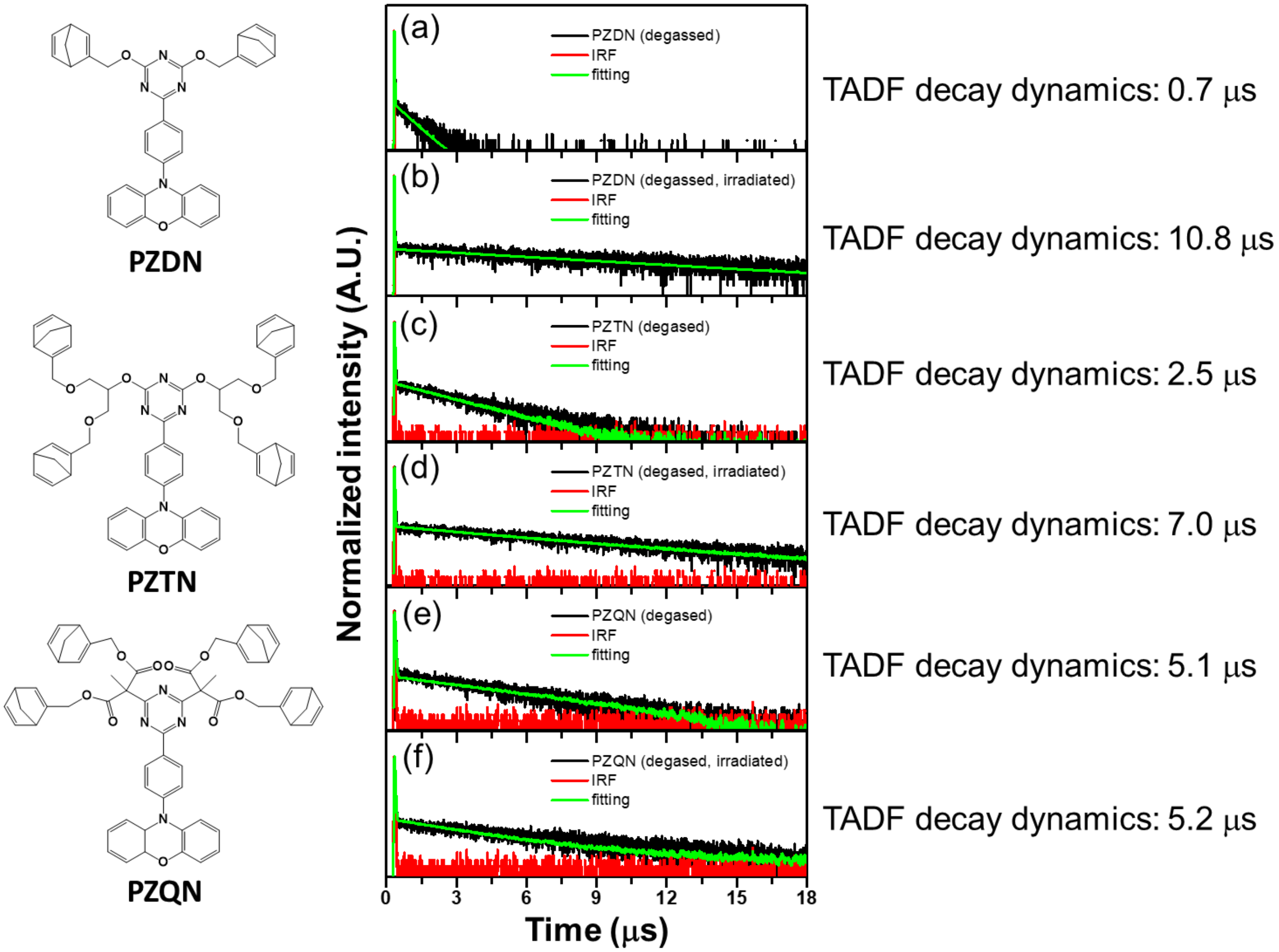

Supplementary Figure 17. The kinetic traces of (a) PZDN, (b) PZDN (after irradiation), (c) PZTN, (d) PZTN (after irradiation), (e) PZQN, and (f) PZQN (after irradiation) in the cyclohexane (in RT). The (**b**) and (**d**) were measured after the complete conversion of NBD to QC. The (**f**) was measured after irradiation of 3 days. λ_{ex} = 405 nm.

The excited state-dynamics is based on thermally activated delayed fluorescence coupled with energy transfer as given in Supplementary Fig. 18. Kinetic rates regarding radiative plus non-radiative decay for the lowest singlet and triplet state, energy transfer of triplets from PXZ-TRZ to NBD moiety, intersystem crossing (S_1 to T_1), and reverse intersystem crossing (T_1 back to S_1) are labeled with k_F, k_P, k_{ET}, k_{ISC}, and k_{RISC}, respectively. Numerical analysis on five rate constants was performed with *Wolfram Mathematica 10.1*[1] by solving the ordinary differential equation followed by optimized non-linear fitting. The generated bi-exponential decay curves accord to the deconvolution fit from TCSPC measurements.

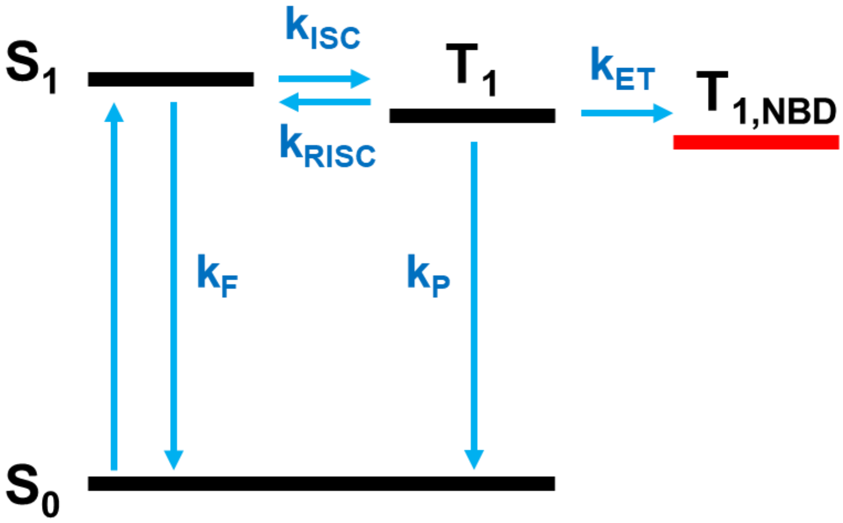

Supplementary Figure 18. The energy transfer was proposed to couple the TADF dynamics in titled molecules. Vertical radiative and non-radiative transition rates were summed into one term, namely, $\mathbf{k_F}$ and $\mathbf{k_P}$ for convenience.

The rate constants were simulated based on the following equations[2,3]. We start from accessing the total decay of S_1 state, which a valid initial guess is given by Supplementary equation (1) - (3). Additionally, k_P is fixed as 10^3 s^{-1} throughout the process.

$$k^{S_1^*} = k_F + k_{ISC} \qquad \text{Supplementary equation (1)}$$

$$k^{T_1^*} = k_P + k_{RISC} \qquad \text{Supplementary equation (2)}$$

$$k_{prompt} \approx k^{S_1^*} \qquad \text{Supplementary equation (3)}$$

where k^{S1^*} and k^{T1^*} refer to the total decay pathway of S_1 and T_1. With equation (S1.3) as the initial condition, we performed iterative, non-linear fitting on k_F, k_{ISC} and k_{RISC} through Supplementary equations (4) - (6).

$$k_{prompt} = \frac{\left(k^{S_1^*} + k^{T_1^*}\right)^2 + \sqrt{\left(k^{S_1^*} + k^{T_1^*}\right)^2 - 4\left(k_F k_P + k_{ISC} k_P + k_{RISC} k_F\right)}}{2} \qquad \text{Supplementary equation (4)}$$

$$k_{delay} = \frac{\left(k^{S_1^*} + k^{T_1^*}\right)^2 - \sqrt{\left(k^{S_1^*} + k^{T_1^*}\right)^2 - 4\left(k_F k_P + k_{ISC} k_P + k_{RISC} k_F\right)}}{2} \qquad \text{Supplementary equation (5)}$$

$$\frac{A_{prompt}}{A_{delay}} = \frac{k^{S_1^*} - k_{delay}}{k_{prompt} - k^{S_1^*}} \qquad \text{Supplementary equation (6)}$$

where k_{prompt}(k_{delay}) and A_{prompt}(A_{delay}) are the rate constant and pre-exponential factor associated to the fast(slow) population decay obtained from the fitting result of TCSPC. Note that equation (S2.3) is added here to optimize the simulated turning point from the prompt to decay region.

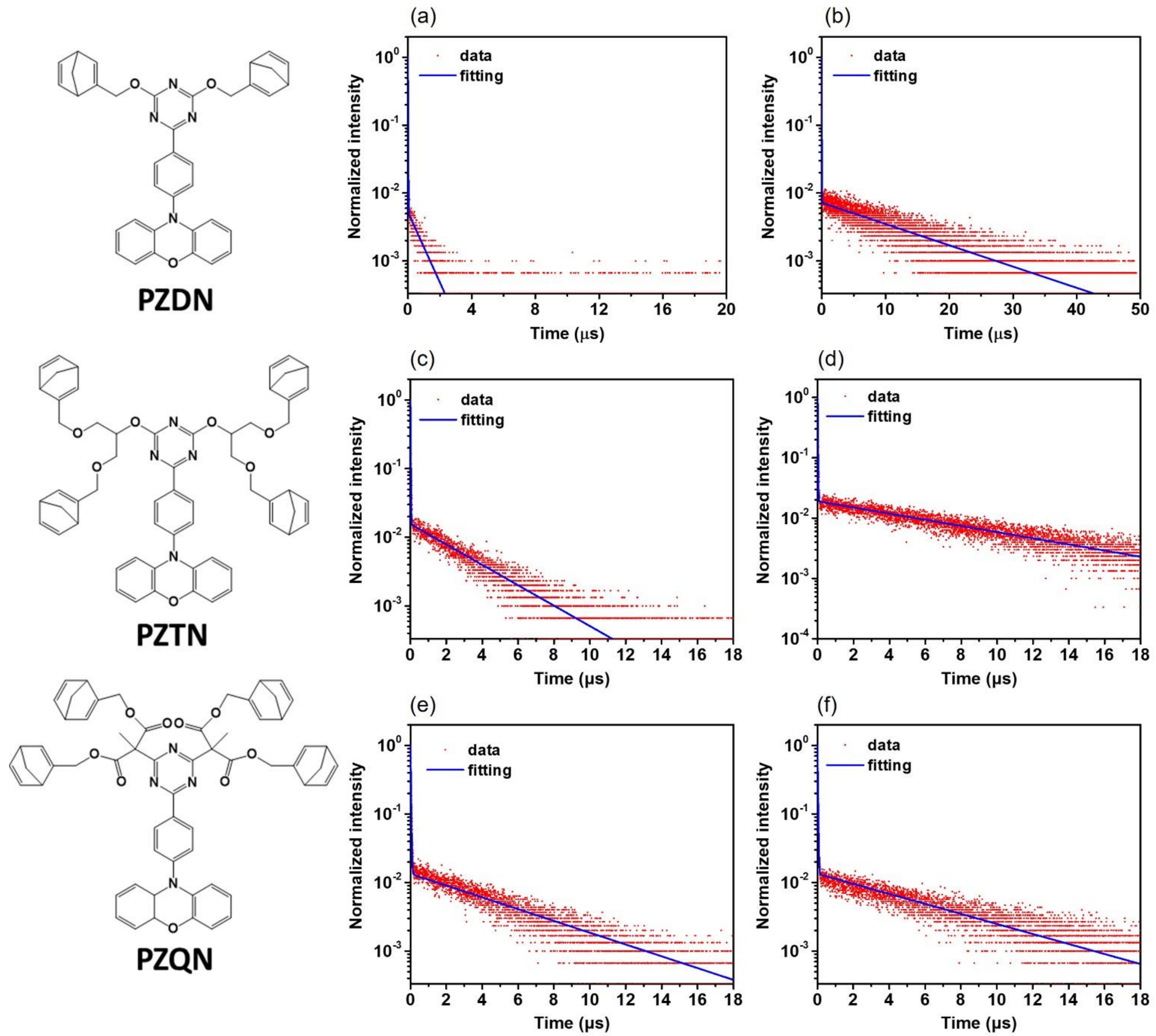

Supplementary Figure 19. Nonlinear fitting curves (blue, solid line) to the TCSPC data (red dot) generated from five optimized rate constants. The decay lifetimes and pre-exponential factors of the simulated decay curves coincide well with the deconvolution fitting of TCSPC results (confer Supplementary Fig.15). The prompt decays from data points are seemingly longer than simulated ones due to the convolution of excitation light and exponential decay for TCSPC measurements. (**a**), (**c**), (**e**) represent PZDN, PZTN, PZQN in degassed condition, respectively, followed by providing sufficient irradiation of 405 nm light giving (**b**), (**d**), (**f**).

Supplementary Table 1. Optimized rate constants extracted from nonlinear fitting curves in Supplementary Fig. 19.

	**Condition**	**k_F** (s^{-1})	**k_P** (s^{-1})	**k_{ET}** (s^{-1})	**k_{ISC}** (s^{-1})	**k_{RISC}** (s^{-1})
**PZDN**	(a) degassed	1.2×10^7	$\approx 10^3$	1.1×10^6	1.2×10^8	7.5×10^5
	(b) degassed+irradiation	9.3×10^6			1.6×10^8	1.3×10^6
**PZTN**	(c) degassed	5.5×10^6		2.5×10^5	8.4×10^7	1.5×10^6
	(d) degassed+irradiation	5.5×10^6			8.2×10^7	1.8×10^6
**PZQN**	(e) degassed	9.9×10^6		3.1×10^4	3.7×10^7	8×10^5
	(f) degassed+irradiation	9.9×10^6			3.7×10^7	8×10^5

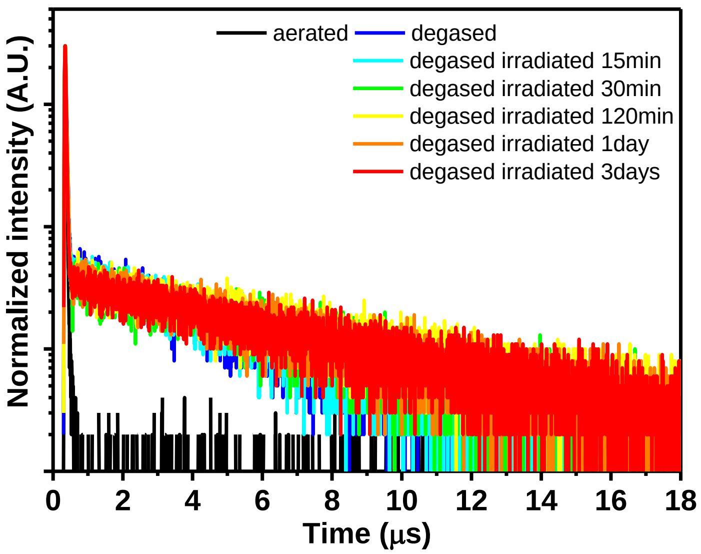

Supplementary Figure 20. The TADF decay dynamics of PZQN as a function of the irradiation time in the room degassed cyclohexane. λ_{ex} = 405 nm.

2-4. Phosphorescence Spectroscopic Measurement (77K):

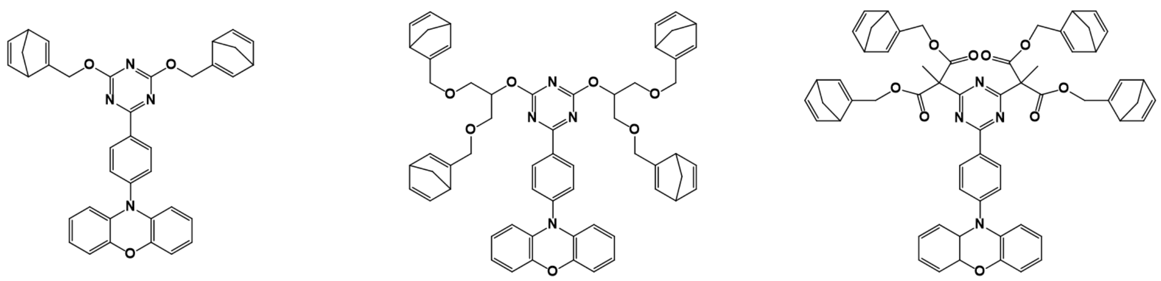

PZDN **PZTN** **PZQN**

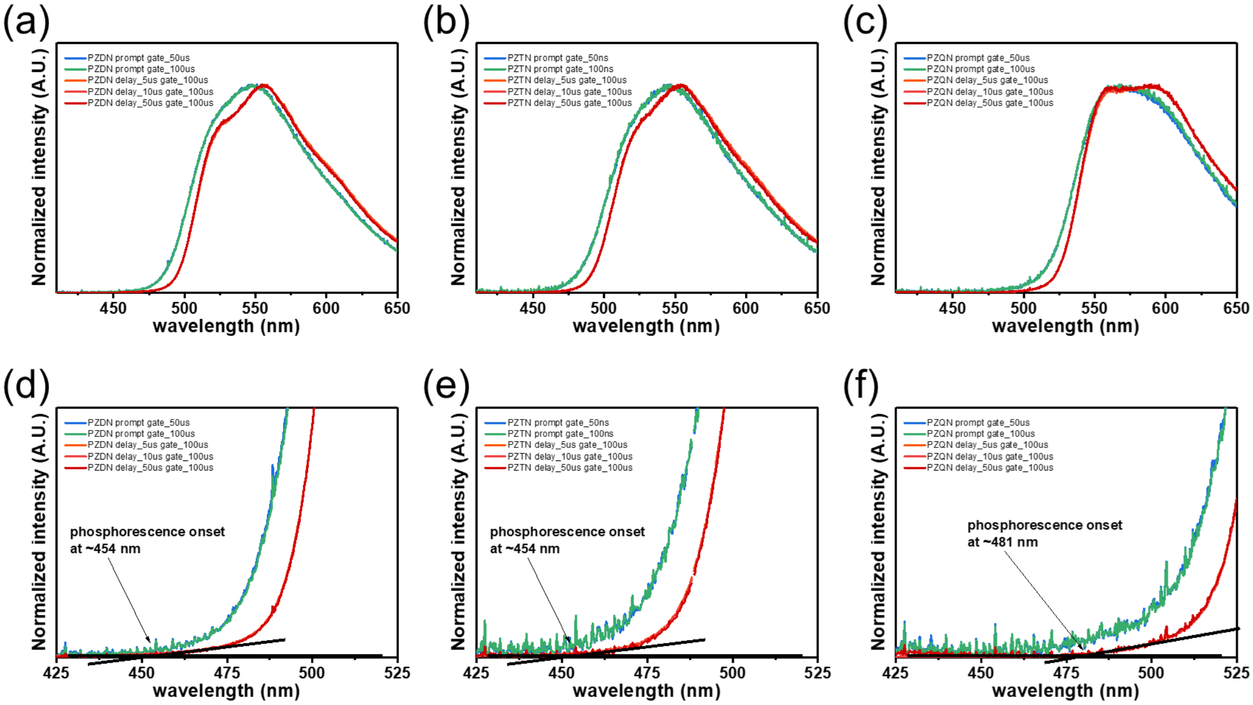

Supplementary Figure 21. The photoluminescence spectra of (a) PZDN, (b) PZTN, (c) PZQN in cyclohexane at 77K, and their phosphorescence onset wavelength are clearly shown in (d), (e), and (f), respectively. “prompt” and “delay_10μs” refers to the starting time of photon collection immediately or 10μs right after the time excitation pulse(λ_{ex} = 370 nm) arrived, respectively. “gate_100μs” indicates the total time of photon collection.

3. Photochemistry and Isomerization

We were preparing a photoisomerization measurement of PZDN. First, the PZDN was dissolved with cyclohexane-d_{12}, then degassed and transferred into a valved NMR tube to prevent influences from O_2. After that, the NMR tube was irradiated in different minutes by a metal halide lamp.

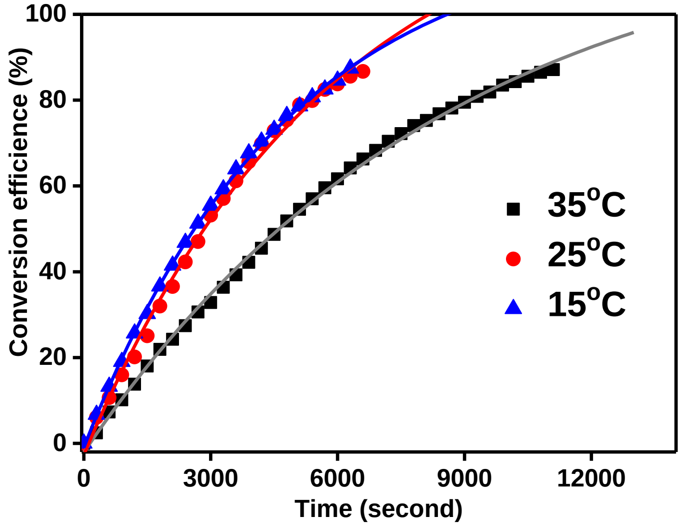

Supplementary Figure 22. Rates of photoisomerization of PZDN in C_6D_{12} at different temperature.

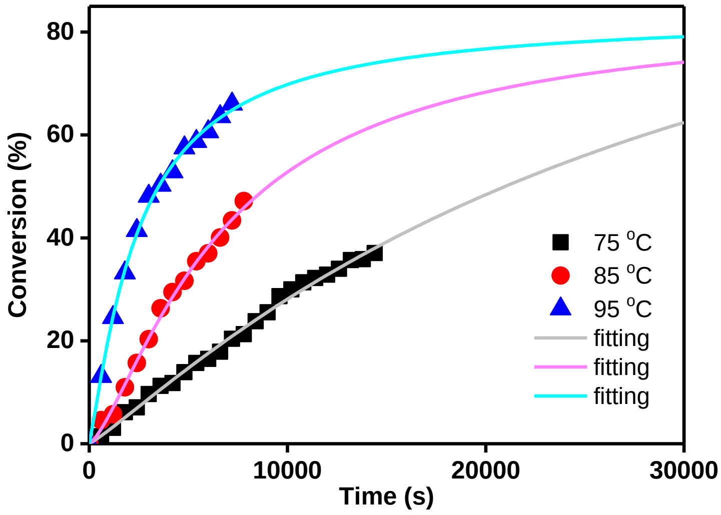

Supplementary Figure 23. Rates of back-conversion of PZDQC in Toluene-d_8 at different temperature.

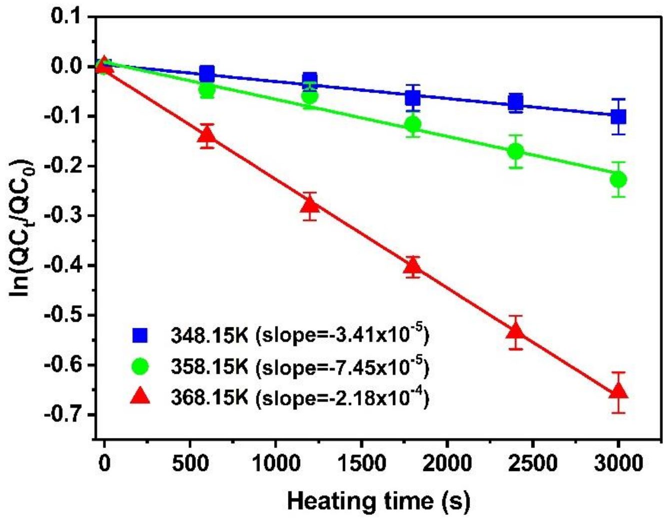

Supplementary Figure 24. The plot of ln (NMR integral variation of PZDQC) versus reaction time in Toluene-d8 at various temperatures.

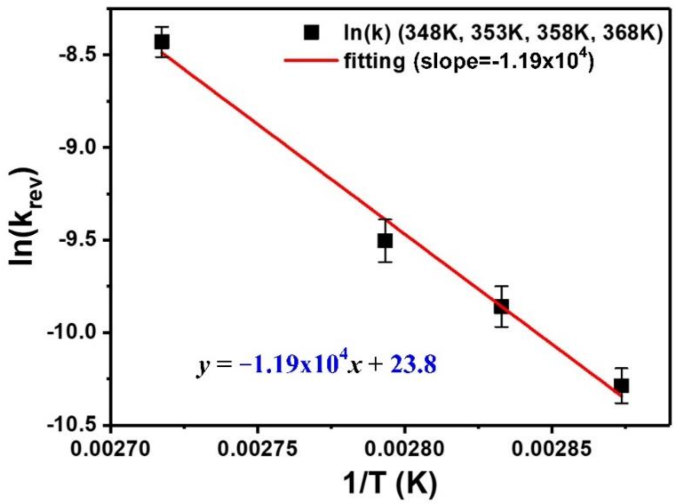

Supplementary Figure 25. The linear plot of ln k_{rev} versus 1/T of PZDQC.

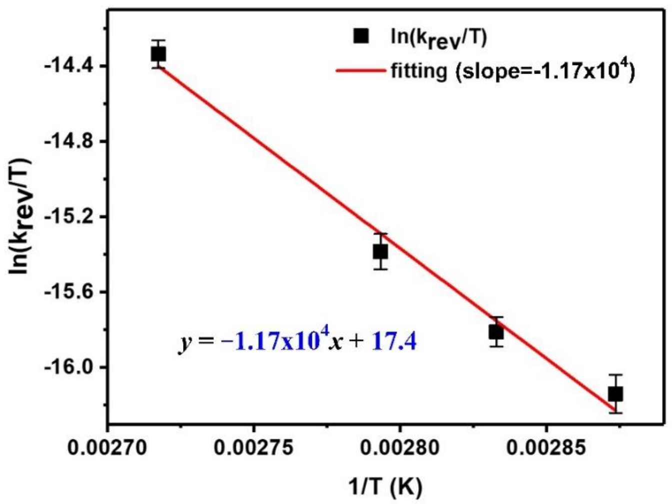

Supplementary Figure 26. The linear plot of ln (k_{rev}/T) versus 1/T.

The reverse reaction for PZDQC and PZTQC with cobalt tetraphenylporphyrin (CoTPP) as catalyst: Dissolved PZDQC or PZTQC in toluene (5 mL), then CoTPP (1 mg) was added and the mixture was stirred at room temperature for 4 h. After that, the solvent was removed in vacuo and the residue was chromatographed on silica gel to afford corresponding PZDN or PZTN in quantitative yield. NMR spectra were identical as shown in Supplementary Fig. 27, 28.

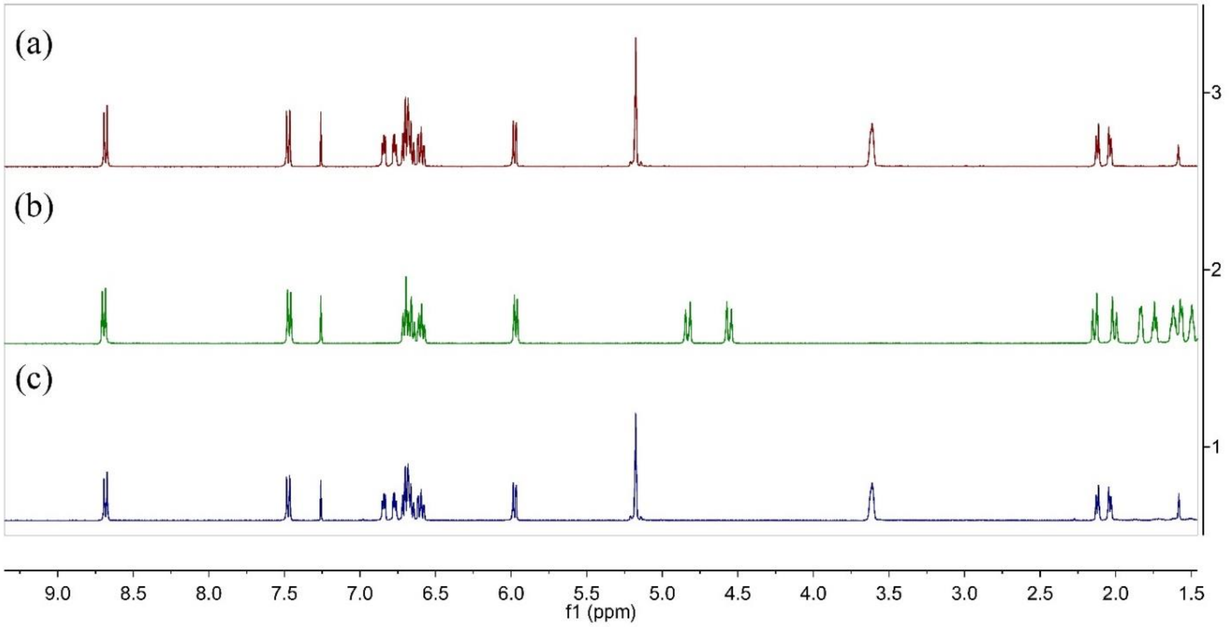

Supplementary Figure 27. The NMR spectra of (a) PZDN, (b) PZDQC and (c) product generated from CoTPP catalyzed PZDQC reverse reaction. (The CoTPP is removed by Chromatography)

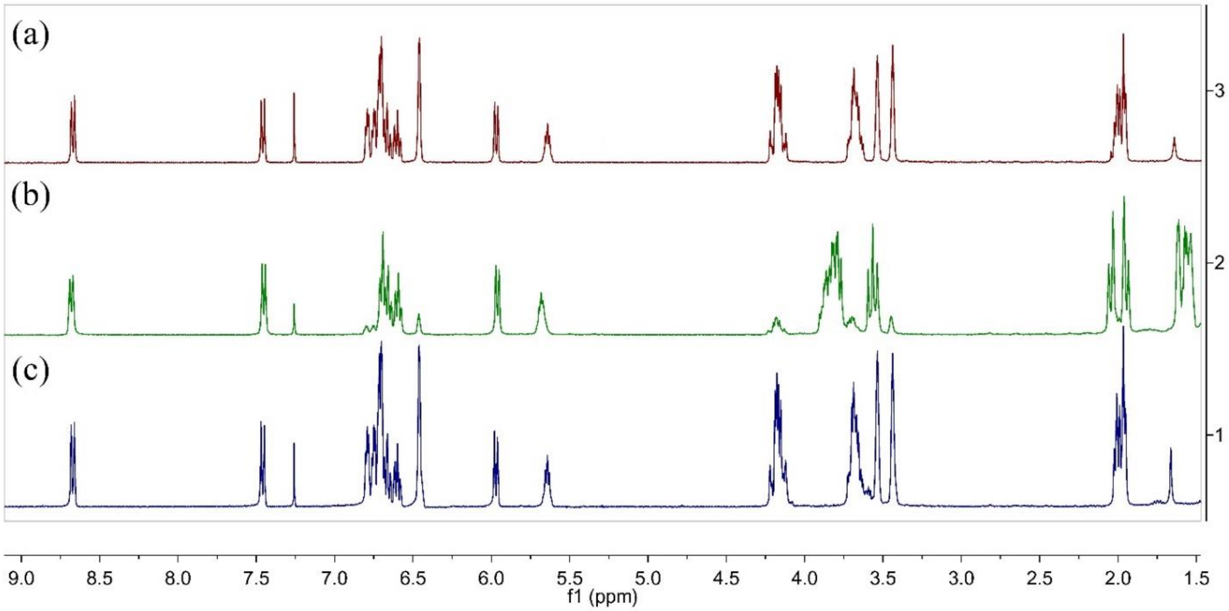

Supplementary Figure 28. The NMR spectra of (a) PZTN, (b) PZTQC and (c) product generated from CoTPP catalyzed PZTQC reverse reaction. (The CoTPP is removed by chromatography)

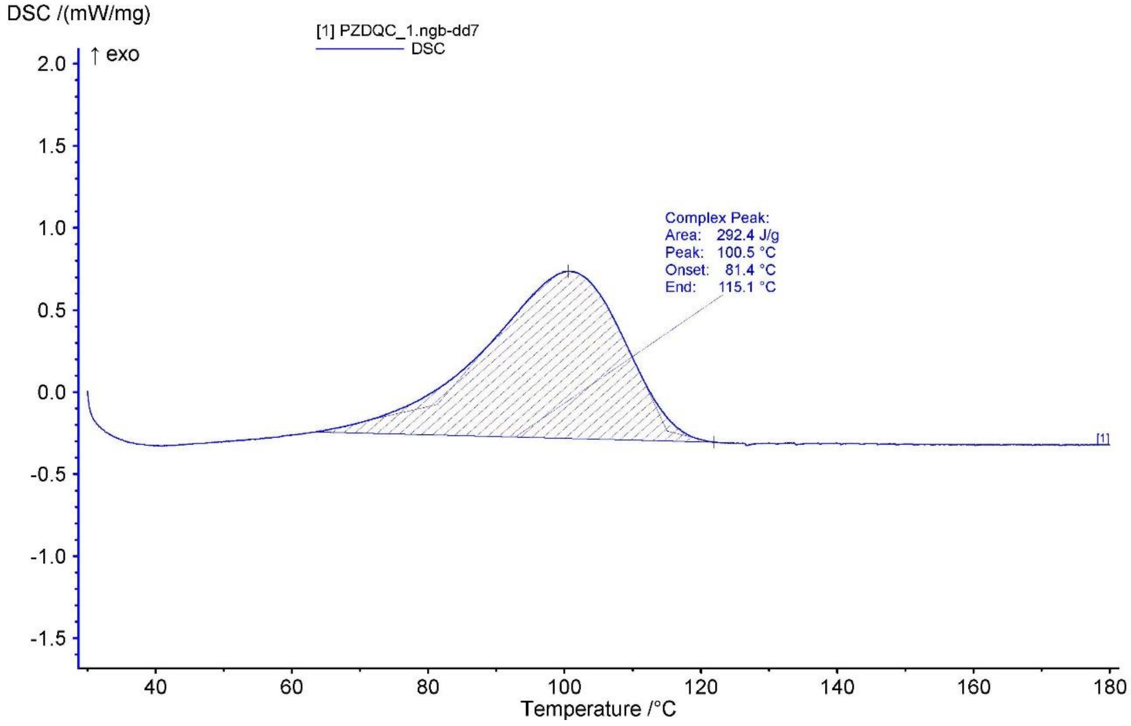

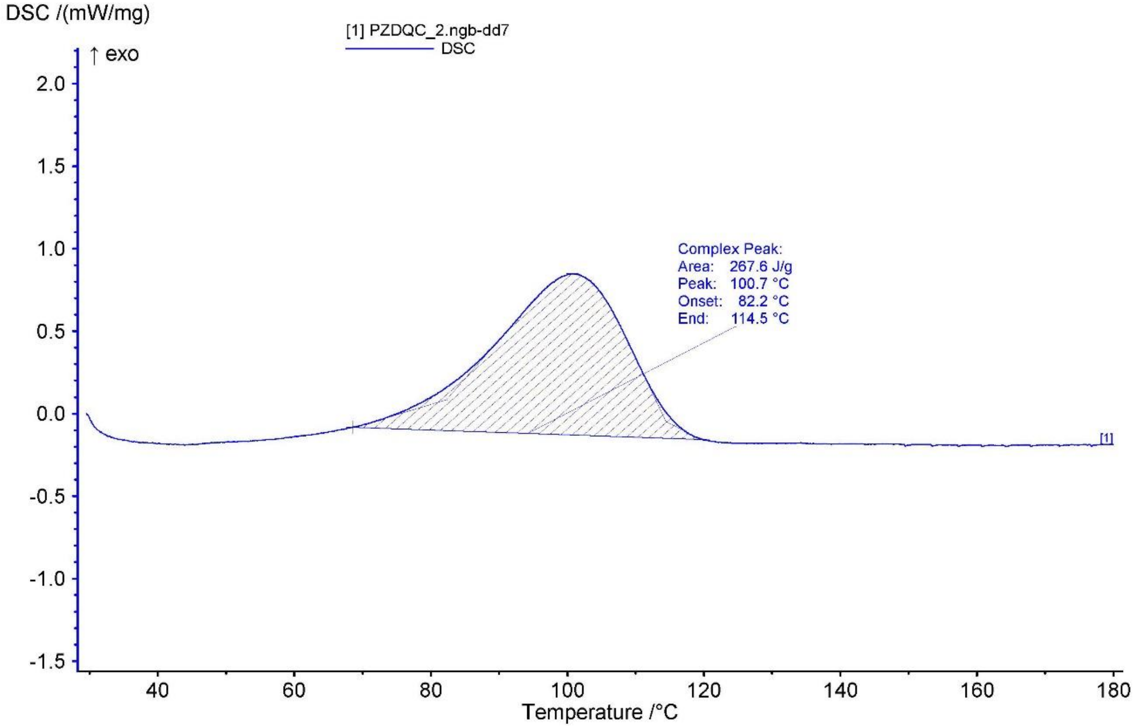

Supplementary Figure 29. DSC thermograms shows the heat release peaks for the thermal back-conversion of PZDQC to the corresponding PZDN. The measurement was repeated twice.

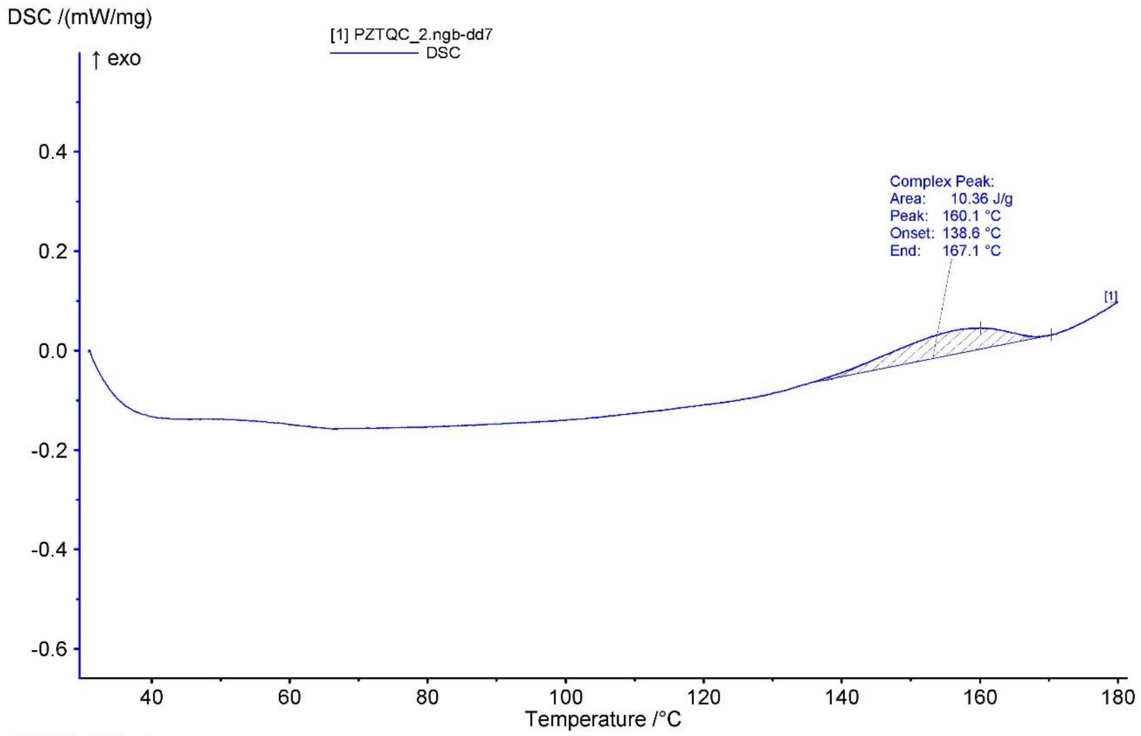

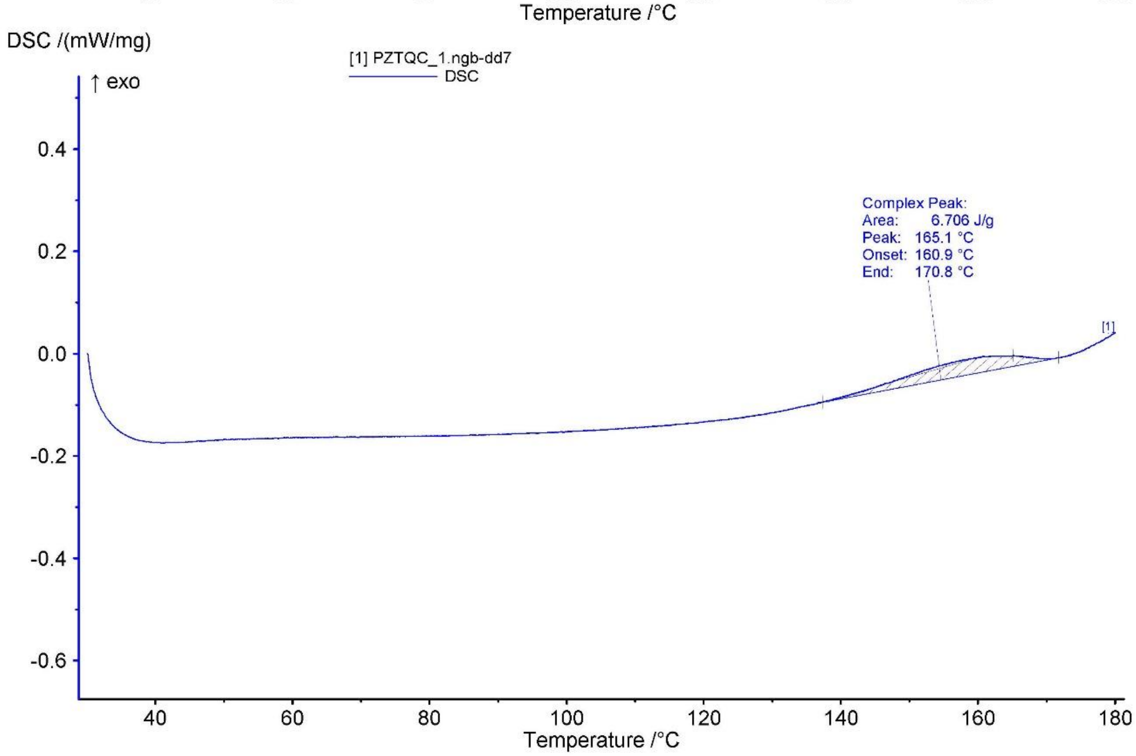

Supplementary Figure 30. DSC thermograms shows the heat release peaks for the thermal back-conversion of PZTQC to the corresponding PZTN. The measurement was repeated twice.

4. Photophysical Measurements in Solid State

We have conducted independent photo-conversion experiments on PZDN in pure solid state (solid film) or imbedded in polymer films including polyethylene(PE), polystyrene(PS), polyvinyl chloride(PVC) or poly(methyl acrylate)(PMMA). Unfortunately, all tested films suffer from severe intermolecular aggregation even in low doping concentration of even less than 0.1 wt%, as indicated by the mismatches of excitation spectra (monitored at different emission wavelength) examined for PZDN@PS film (~0.1 wt%) shown below.

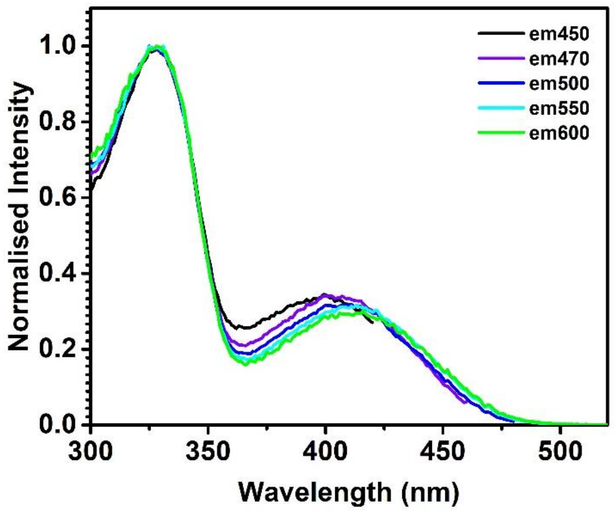

Supplementary Figure 31. Excitation spectra of PZDN@PS (< 0.1 wt%). Note that “em 450” refers to the monitored emission wavelength at 450 nm.

As shown in Supplementary Fig. 31, we observe a red-shifted excitation spectrum upon monitoring at 600 nm emission, compared to that when monitoring the emission at 450 nm. The emission wavelength dependent excitation spectra render evidence of intermolecular interactions and thus possible aggregation. This aggregation effect is more significant in high concentration polymer films and pure PZDN powder. The molecular aggregation results in lowering the triplet state energy, which is unfortunately lower than that of the NBD’s triplet state, prohibiting the Dexter-type triplet-triplet energy transfer efficiency. As a result, we are unable to observe the changes in population lifetime (monitored at emission peak) upon irradiating 405 nm-light in pure solid PZDN film, PZDN doped PE film or PMMA film, where the solubility of PZDN is low and aggregation becomes dominant. As for doping PZDN in PVC and PS films, the presence of aggregation seems to be suppressed and there is still a small portion of photo-conversion for PZDN monomer, as implied by the increase of population lifetime during the photolysis. The corresponding kinetic traces and emission spectra as a function of irradiation time are shown in Supplementary Fig. 32 and 33.

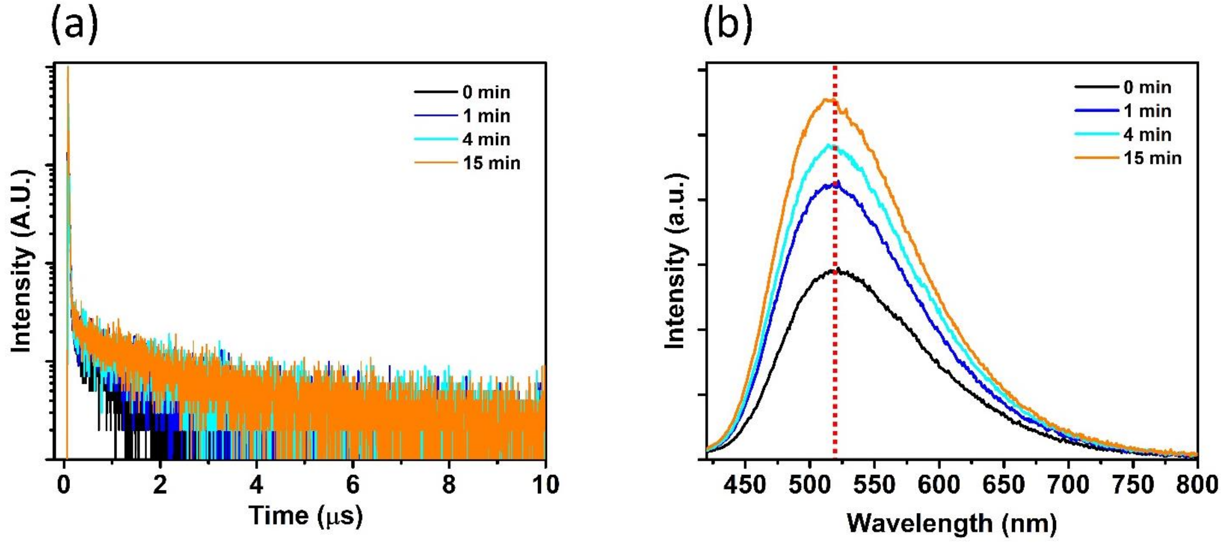

Supplementary Figure 32. (a) Kinetic trace and (b) emission spectra of PZDN@PVC (0.1 wt%) as a function of 405 nm-irradiation time.

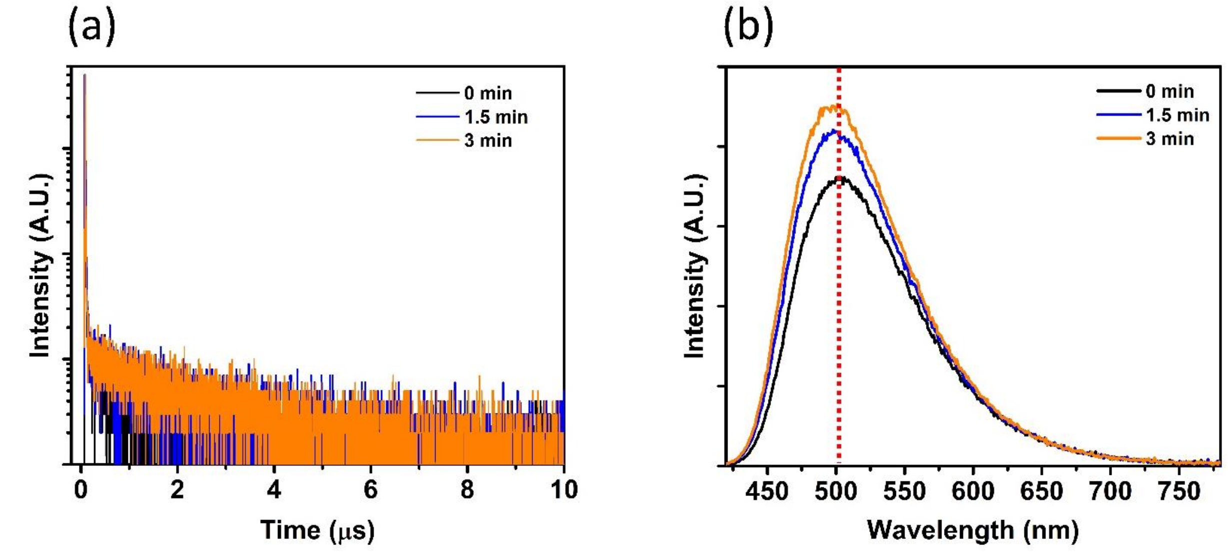

Supplementary Figure 33. (a) Kinetic trace and (b) emission spectra of PZDN@PS (0.1 wt%) as a function of 405 nm-irradiation time.

Clearly, in both PVC and PS hosts, the emission spectrum gradually blue shifts due to the PZDN→PZDQC conversion of monomer. Additionally, PZDN@PVC has a better conversion efficiency than in PS, indicating more homogeneous dispersion of PZDN in PVC film. Furthermore, we managed to access the reversibility between PZDN and PZDQC in both PVC and PS through irradiating the 405 nm light (3 W LED lamp, 15 minutes to ensure full conversion of PZDN→PZDQC, marked as red dot and red line in Supplementary Fig. 34, 35) and heating at 95°C in a clean oven for an hour (back-conversion of PZDQC→PZDN, marked as blue dot and blue line in Supplementary Fig. 34, 35). The results of photo-thermal conversion cycles are shown in Supplementary Fig. 34 and 35 for PZDN@PVC and PZDN@PS, respectively.

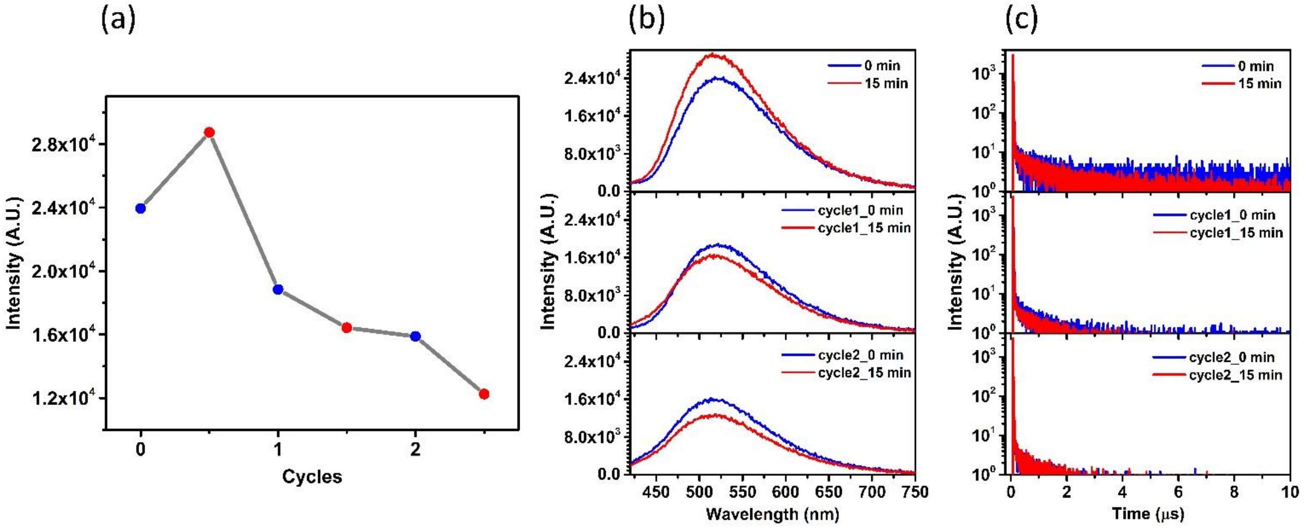

Supplementary Figure 34. The durability test for PZDN@PVC (●) ⇆ PZDQC@PVC (●) (0.1 wt%). (a) Emission intensity monitored at 520 nm. The corresponding (b) emission profiles and (c) population decays during the photo-thermal conversion cycles 0, 1 and 2.

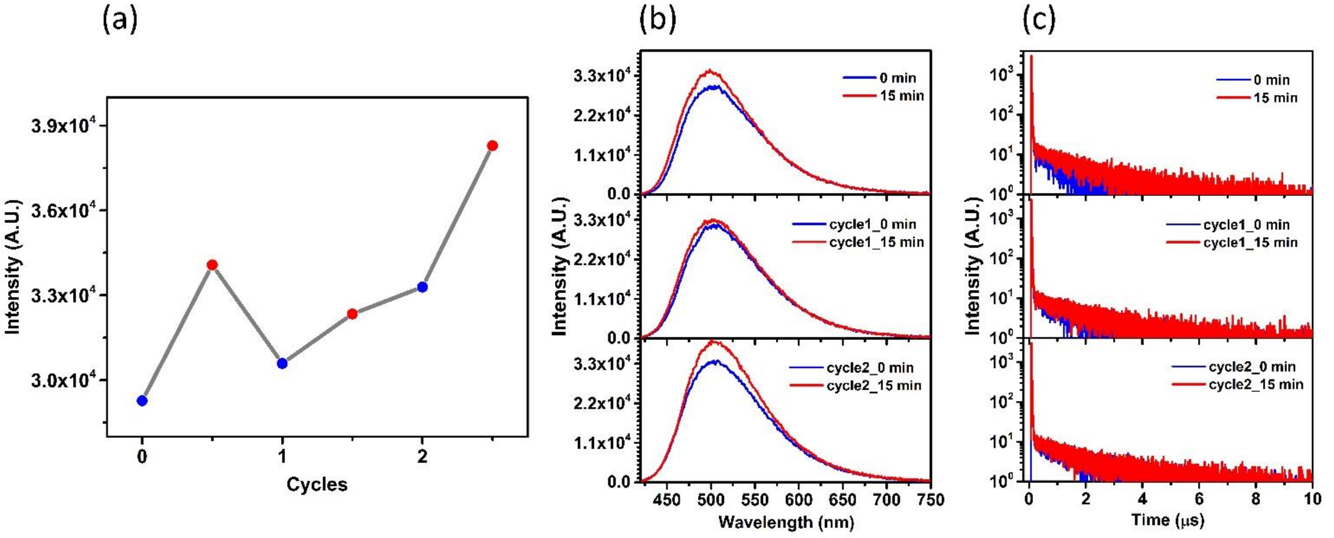

Supplementary Figure 35. The durability test for PZDN@PS (●) ⇆ PZDQC@PS (●) (0.1 wt%). (a) Emission intensity monitored at 500 nm. The corresponding (b) emission profiles and (c) population decays during the photo-thermal conversion cycles 0, 1 and 2.

Plot (a) of Supplementary Fig. 34 and 35 shows the intensity monitored at emission peak, while (b) and (c) refer to the corresponding emission spectra and decay profile in the photo-thermal conversion cycles 0, 1 and 2. The results show that PZDN in PVC solid films, albeit good photo-conversion efficiency at the first cycle, suffers from severe aggregation and polymerization[4] during the next light irradiation and heating, resulting in a dramatic decrease in emission intensity and shortening in the decay kinetic trace. As for PZDN@PS, the trend in emission intensity holds for 3 cycles (increases after irradiation, decreases after heating), which again verifies PS to be a more suitable host for NBD/QC system[5]. Note that the anomalous increase in intensity (2nd red dot to 3rd blue dot) is caused by the inhomogeneous thickness of the film, where the steady state measurements for each cycle deviates from the focused excitation source.

5. Supplementary References

1. Wolfram Research, Inc., *Mathematica, Version 10.1*, Champaign, (2015).456

2. Goushi, K., Yoshida, K., Sato, K. & Adachi, C. Organic Light-emitting Diodes Employing Efficient Reverse Intersystem Crossing for Triplet-to-Singlet State Conversion. *Nature Photon.* **6,** 253–258 (2012).

3. Tsuchiya, Y., Diesing, S., Bencheikh, F., Wada, Y., dos Santos, P. L., Kaji, H., Zysman-Colman, E., Samuel, I. D. W. & Adachi, C. Exact Solution of Kinetic Analysis for Thermally Activated Delayed Fluorescence Materials. *J. Phys. Chem. A* **125**, 8074-8089 (2021).

4. Dreos A., Wang, Z., Udmark, J., Ström, A., Erhart, P., Börjesson, K., Nielsen, M. B. & Moth-Poulsen, K. Liquid Norbornadiene Photoswitches for Solar Energy Storage. *Adv. Energy Mater.* **8**, 1703401 (2018).

5. Petersen, A. U., Hofmann, A. I., Fillols, M., Mansø, M., Jevric, M., Wang, Z., Sumby, C. J., Müller, C. & Moth-Poulsen, K. Solar Energy Storage by Molecular Norbornadiene–Quadricyclane Photoswitches: Polymer Film Devices, *Adv. Sci.* **6**, 1900367 (2019).
